# Syphilis-Related Nephropathy: A Rare Manifestation of a Re-emerging Disease

**DOI:** 10.7759/cureus.50105

**Published:** 2023-12-07

**Authors:** Aya Aal Hamad, Zeyana Al Hadhrami, Ali Al Lawati, Ibrahim Al Busaidi, Saja Mahmood

**Affiliations:** 1 Emergency Medicine, Sultan Qaboos University Hospital, Muscat, OMN; 2 Medicine, Sultan Qaboos University Hospital, Muscat, OMN

**Keywords:** c3 glomerulonephritis, hiv, post-infectious glomerulonephritis, secondary membranous nephropathy, syphilis

## Abstract

Syphilis is a curable sexually transmitted infection caused by the spirochete *Treponema pallidum*. Its clinical manifestations are variable as it has a remarkable aptitude to imitate a spectrum of clinical pictures. This phenomenon has bestowed upon it the epithet "the great imitator" within the medical literature. The escalating global prevalence of syphilis cases underscores the importance of shedding light on its rare manifestations. Syphilitic nephropathy is an uncommon manifestation of secondary syphilis. Here, we report two cases of syphilis-related nephropathy, the first presented as a nephrotic syndrome, and the second as a nephritic syndrome. Both cases had a favorable outcome after treatment of syphilis with benzathine penicillin G.

## Introduction

Syphilis is a systemic disease caused by a motile Gram-negative bacterium, *Treponema pallidum*. It has witnessed an increasing incidence across multiple countries, including the Americas, Europe, China, New Zealand, and Australia since the year 2000, mainly among males who have sex with males [1-4]. The variable clinical presentations of syphilis and the feasibility of its cure necessitate considering such diagnosis in high-risk patients. The prevalence of kidney involvement in secondary syphilis varies between 0.3% and 0.8% [5]. The diagnosis requires a high level of suspicion in high-risk patients. It is often misdiagnosed due to its variable and rare clinical manifestations. The disease is categorized into early and late stages depending on the estimated time since infection and the clinical features. Early infection encompasses primary, secondary, and early latent syphilis, while late stages encompass late latent and tertiary syphilis. Primary syphilis is marked by the development of genital or oral ulcers, known as chancres, within a few weeks of exposure. Secondary syphilis occurs 4-10 weeks after exposure and manifests as a maculopapular rash, adenopathy, hepatitis, and constitutional symptoms. Renal involvement is a rare manifestation of secondary syphilis. Tertiary syphilis affects the cardiovascular and neurological systems as well as granulomata formation, known as gummatous syphilis [6-8]. Syphilis has variable renal manifestations. It can present as nephrotic syndrome of which membranous nephropathy is the most common presentation, but it can also manifest as acute kidney injury, membranoproliferative glomerulonephritis, or interstitial nephritis [9,10]. Granulomatous kidney disease (Gumma) has also been described [11]. This study has presented the clinical trajectory of two patients diagnosed with syphilis-related nephropathy. The cases presented herein serve to augment the existing body of knowledge and heighten awareness regarding the diverse clinical presentations of syphilitic nephropathy.

## Case presentation

Case 1

**Figure 1:**
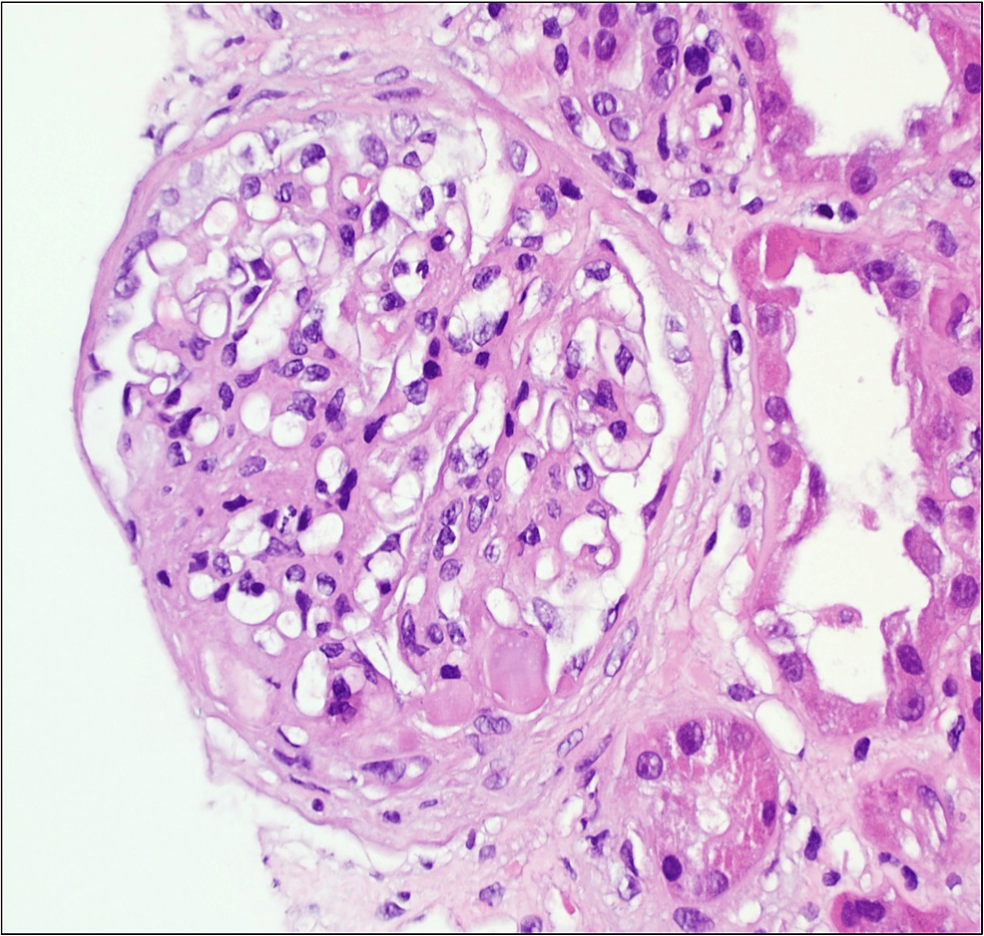
Mesangial expansion by cells and matrix with segmental sclerosis (H&E stain 20x).

**Figure 2:**
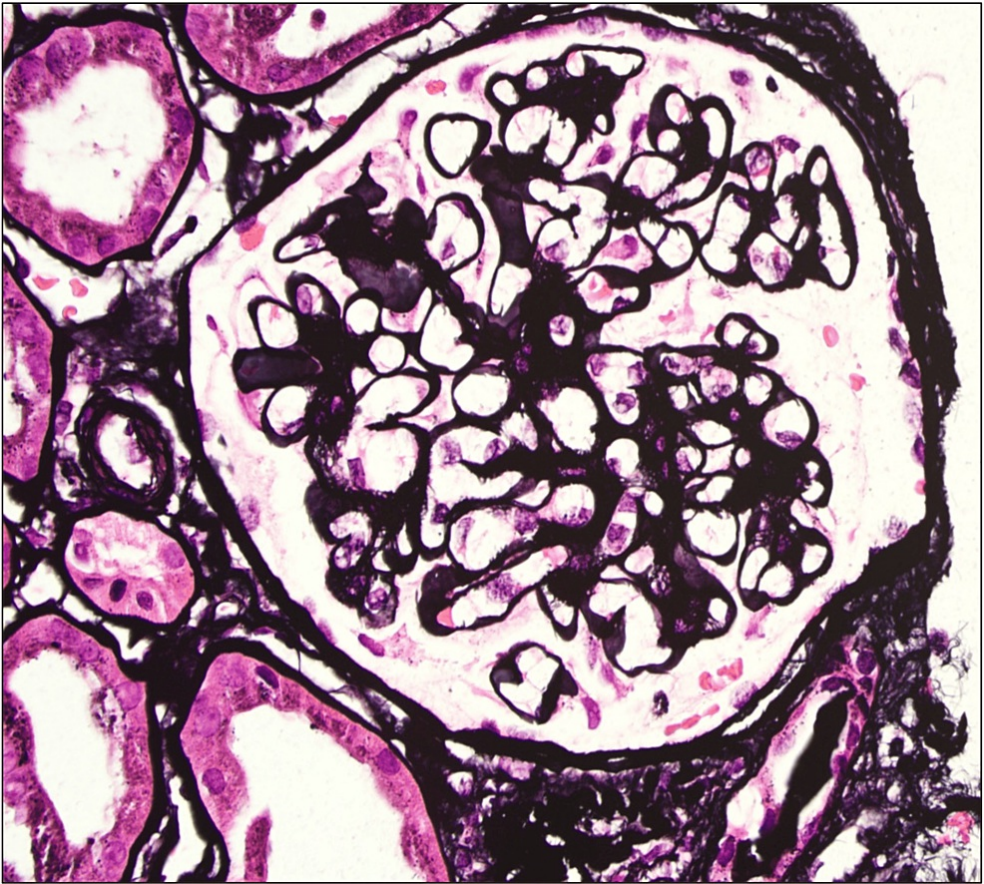
Thickened capillary walls but no evidence of spikes/reticulations or double contours (Jones silver stain 20x).

We present a case of a 26-year-old male with a history of type 1 diabetes mellitus since the age of 12 years. He was diagnosed with human immunodeficiency virus (HIV) infection six months prior to his presentation for which he is on tenofovir alafenamide (TAF), emtricitabine (FTC), and dolutegravir (DTG) with virological suppression. He presented to the emergency department with a new onset of bilateral lower limb swelling and weight gain. His blood pressure, oxygen saturation, and pulse rate were within normal limits. His clinical examination was consistent with volume overload. Laboratory investigations revealed microcytic hypochromic anemia, with a hemoglobin level of 7.7 g/dL. The patient also exhibited significant proteinuria (urine protein-to-creatinine ratio of 2153 mg/mmol) and acute kidney injury (AKI), with a creatinine of 156 umol/L from a baseline of 80 umol/L one month earlier, and an estimated glomerular filtration rate (eGFR) of 46 mL/min/1.73 m^2^. Serum albumin was low at 18 g/L, and 24-hour urine protein collection revealed 18 g/day. Abdominal ultrasound was done and showed normal-size kidneys with no evidence of obstruction. Urine microscope and culture were negative. HIV-associated nephropathy was considered less likely due to the patient's suppressed viral load. His workup for nephrotic syndrome showed normal anti-nuclear antibody (ANA), complement levels, anti-phospholipase A2 receptor (PLA2R) antibodies, serum, and urine protein electrophoresis, and glycated hemoglobin (HbA1c) 7.7%. A kidney biopsy was performed, which revealed diffuse global and segmental glomerulosclerosis, and features of diabetic glomerulosclerosis (Figures 1, 2).

The biopsy also showed moderate interstitial fibrosis and tubular atrophy, affecting approximately 40% of the sample. The immunofluorescence (IF) microscopy demonstrated staining to IgG, lambda, and kappa. His PLA2R antibody staining was negative. There was no tissue submitted for electron microscopy. The finding was suggestive of secondary membranous nephropathy. Additional investigations were carried out to elucidate the underlying cause of membranous nephropathy. Notably, the patient tested positive for syphilis serology, with a rapid plasma regain (RPR) titer of 1:128 and a *T. pallidum* hemagglutination assay (TPHA) titer greater than 1:10240, consistent with secondary syphilis. He was initiated on treatment with three doses of 2.4 million units dose of weekly benzathine penicillin G intramuscular injections for presumed late latent syphilis. To manage his nephropathy and edema, he was started on furosemide and angiotensin receptor blocker. He responded well with significant improvement in his edema, creatinine, and proteinuria. The improvement in his renal parameters correlated well with an appropriate drop in his RPR and TPHA titers.

Case 2

**Figure 3:**
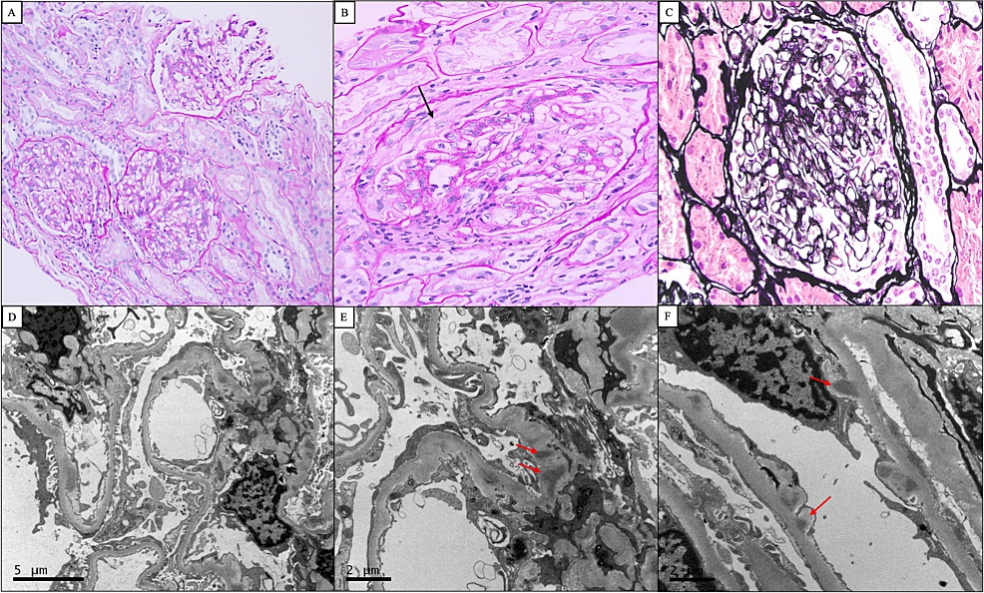
Electron microscopy (EM) showing few mesangial and sub-endothelial deposits. The images show (A) three glomeruli with mesangial hypercellularity and matrix expansion. Tubules in the background appear unremarkable (PAS stain 10x). (B) Higher magnification of one glomerulus shows a fibrocellular crescent (black arrow) (PAS stain 40x). (C) A glomerulus with normal thickness and texture of capillary walls (Jones silver stain 40x). (D) Electron microscopy micrograph shows normal thickness and texture of glomerular basement membranes with partial foot process effacement (7500x). (E) Numerous peripheral mesangial deposits were seen (red arrows) (10000x). (F) Numerous small sub-endothelial deposits were also seen (red arrows) (12000x). PAS: Periodic acid-Schiff

A 32-year-old male with underlying asthma, not on medications, presented to the emergency department with new-onset hypertension, acute bilateral flank pain, and back pain, accompanied by cola-colored urine and a non-pruritic generalized maculopapular rash. Additionally, he had palpable cervical lymphadenopathy. A complete blood count revealed hypochromic microcytic anemia, with a hemoglobin level of 11 g/dL, normal platelets, and white cell count. His creatinine was 188 umol/L and his urine analysis was positive for blood and protein. His urine polymerase chain reaction (PCR) was 350 mg/mmol. His complement levels, ANA, and anti-nuclear cytoplasmic antibodies (ANCA) serology were within normal levels. A kidney biopsy was performed which revealed mesangial proliferative glomerulonephritis along with minimal interstitial fibrosis and tubular atrophy on a light microscope. The immunofluorescence (IF) profile showed positive C3 stating (+1) but negative for IgA, IgG, IgM, C1q, kappa, and lambda which is consistent with C3 glomerulonephritis. Electron microscopy (EM) showed few mesangial and sub-endothelial deposits (Figures 3A-3F).

Given the presence of cervical lymphadenopathy, he underwent a CT scan of the neck, chest, and abdomen that showed sub-centimeter cervical lymph nodes, with the largest one measuring 0.8 cm in the sub-mandibular region. However, the spleen, liver, and kidneys appeared normal. Considering glomerulonephritis and the patient's clinical presentation, a workup for infectious etiologies (post-infectious C3 glomerulonephritis {GN}) was initiated. The patient was screened for syphilis, which showed positive results for *T. pallidum* hemagglutination assay (TPHA) and a rapid plasma reagin (RPR) titer of 1:64, confirming the diagnosis of secondary syphilis. He received a single dose of 2.4 million units of intramuscular benzathine penicillin G. The patient also reported urethral discharge, prompting the investigation of a possible sexually transmitted infection. *Chlamydia trachomatis* infection was confirmed by Gene Xpert PCR for Chlamydia in urine, and appropriate treatment with a seven-day course of doxycycline was initiated as well. His creatinine improved as well as his proteinuria, rash, and lymphadenopathy.

## Discussion

The pathogenesis of syphilis-related nephropathy has been attributed to immune complex deposition through direct identification of *T. pallidum *antigen and antibodies within immune complexes in renal tissue biopsies [12,13]. Membranous nephropathy (MN) is the most common manifestation of secondary syphilis. It is characterized by thickening of the glomerular basement membrane (GBM) due to sub-epithelial immune complex deposition. Primary MN is believed to be due to autoantibodies binding endogenous antigens on the surface of the podocytes. M-type phospholipase A2 receptor (PLA2R) is the target antigen in 80% of primary MN [14,15]. In our first case, PLA2R antibodies were negative in the serum and immune fluorescence on tissue biopsy, suggesting a secondary cause of MN. This triggered thorough investigations with an extensive history including patient demographics, medications, and past exposure to toxic substances, along with tests and imaging studies to identify possible hidden malignancies or infections as a cause of MN. Nevertheless, antibodies against PLA2R have been reported in syphilis-related MN which demonstrates the importance of considering syphilis infection as a cause of MN regardless of PLA2R antibody status [16].

Other glomerular lesions have also been described in the literature including membranoproliferative glomerulonephritis, focal segmental glomerulosclerosis, minimal change disease, and crescentic glomerulonephritis [17-19]. Our patient in case two presented with nephritic syndrome and a classical prodrome of secondary syphilis (rash and adenopathy). His kidney biopsy was consistent with C3 predominant post-infectious glomerulonephritis. His complement levels were normal by the time he had the biopsy. Serum C3 level is usually but not always low in patients with C3 glomerulopathy. Although he also had a co-infection with *C. trachomatis*, there is no reported association in our knowledge between *C. trachomatis* and glomerulonephritis.

**Figure 4:**
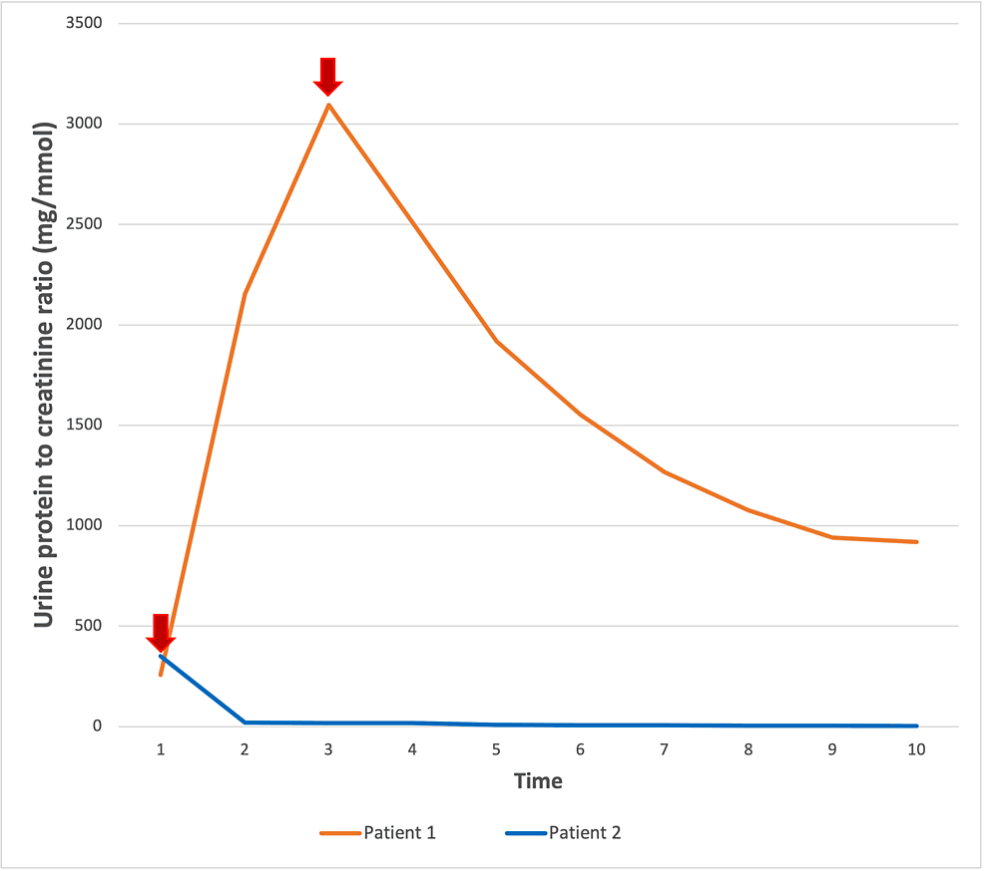
Response of proteinuria to the initiation of intramuscular (IM) benzathine penicillin G in both patients. The arrows represent the time of treatment initiation.

**Figure 5:**
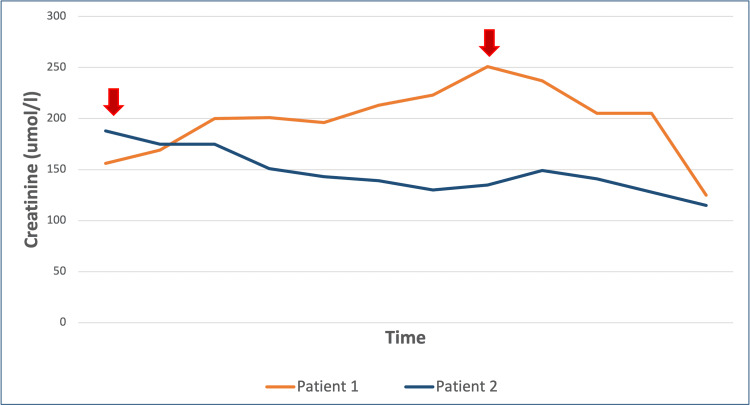
Creatinine response to the initiation of intramuscular (IM) benzathine penicillin G in both patients. The arrows represent the time of treatment initiation.

The management of syphilis in both cases resulted in a significant improvement in kidney function. The improvement might be less dramatic in the first patient likely because of his background of long-standing type 1 diabetes with evidence of diabetic nephropathy with interstitial fibrosis and tubular atrophy that was evident in his kidney biopsy (Figures 4, 5).

## Conclusions

Syphilis-related nephropathy, a rare renal manifestation of syphilis infection, needs to be considered in high-risk patients as it might be the only manifestation of the disease. The reported two cases emphasize the significance of diagnosing and managing syphilis infection for better patient outcomes.

## References

[1] Kojima N, Klausner JD (2018). An update on the global epidemiology of syphilis. Curr Epidemiol Rep.

[2] Spiteri G, Unemo M, Mårdh O, Amato-Gauci AJ (2019). The resurgence of syphilis in high-income countries in the 2000s: a focus on Europe. Epidemiol Infect.

[3] Tao Y, Chen MY, Tucker JD (2020). A nationwide spatiotemporal analysis of syphilis over 21 years and implications for prevention and control in China. Clin Infect Dis.

[4] Tsuboi M, Evans J, Davies EP (2021). Prevalence of syphilis among men who have sex with men: a global systematic review and meta-analysis from 2000-20. Lancet Glob Health.

[5] Hunte W, al-Ghraoui F, Cohen RJ (1993). Secondary syphilis and the nephrotic syndrome. J Am Soc Nephrol.

[6] Ghanem KG, Ram S, Rice PA (2020). The modern epidemic of syphilis. N Engl J Med.

[7] Aral SO, Over M, Manhart L, Holmes KK (2006). Sexually transmitted diseases. Disease Control Priorities in Developing Countries. Second Edition.

[8] Purwoko MI, Nugroho SA, Pamudji R, Devi M, Fitriani F, Candra NC (2021). Laboratory examination of syphilis. J Biomed Transl Res.

[9] Shettigar R, Schollum J, Putt T, Chan L, Lau M, Walker R (2021). Renal manifestations of syphilis. Intern Med J.

[10] Zhang Z, Hever A, Bhasin N, Kujubu DA (2018). Secondary syphilis associated with membranous nephropathy and acute hepatitis in a patient with HIV: a case report. Perm J.

[11] de Carvalho JG, Slongo EL, Sobral AC (2007). Kidney mass and osteolytic lesion: is it always malignancy?. Nephrol Dial Transplant.

[12] Tourville DR, Byrd LH, Kim DU, Zajd D, Lee I, Reichman LB, Baskin S (1976). Treponemal antigen in immunopathogenesis of syphilitic glomerulonephritis. Am J Pathol.

[13] Gamble CN, Reardan JB (1975). Immunopathogenesis of syphilitic glomerulonephritis. Elution of antitreponemal antibody from glomerular immune-complex deposits. N Engl J Med.

[14] Liu W, Gao C, Dai H (2019). Immunological pathogenesis of membranous nephropathy: focus on PLA2R1 and its role. Front Immunol.

[15] Beck LH Jr, Bonegio RG, Lambeau G (2009). M-type phospholipase A2 receptor as target antigen in idiopathic membranous nephropathy. N Engl J Med.

[16] Wanderley DC, Neves PD, Jorge LB, Onuchic LF, Araujo SA (2021). The immunohistological profile of membranous nephropathy associated with syphilis infection. Clin Kidney J.

[17] Tognetti L, Cinotti E, Tripodi S, Garosi G, Rubegni P (2018). Unusual presentation of secondary syphilis: membranoproliferative glomerulonephritis andmuco-cutaneous lesions. Int J STD AIDS.

[18] Hartley AJ, Rajakariar R, Sheaff M, Buckland M, Goh B, O'Connell R (2014). Syphilis masquerading as focal segmental glomerulosclerosis. Int J STD AIDS.

[19] Walker PD, Deeves EC, Sahba G, Wallin JD, O’Neill WM Jr (1984). Rapidly progressive glomerulonephritis in a patient with syphilis. Identification of anti-treponemal antibody and treponemal antigen in renal tissue. Am J Med.

